# Exploration of Antioxidative, Antidiarrheal, and Antihyperglycemic Properties of *Artocarpus chama* Leaves Along With In Silico Analysis

**DOI:** 10.1155/bmri/9930195

**Published:** 2025-01-28

**Authors:** Md. Shahriar Hossen, K. M. Nazmul Hasan Refat, Muaz Faruque, Pritam Kundu, Utpal Kumar Karmakar

**Affiliations:** Pharmacy Discipline, Life Science School, Khulna University, Khulna, Bangladesh

## Abstract

*Artocarpus chama* is a rare member of the Moraceae family that is found in some Asian countries. This plant has some therapeutic uses in folk medicine. This project was carried out on the leaves of this plant to explore some of its pharmacological importance. This plant revealed the presence of different phytochemical groups such as reducing sugars, tannins, flavonoids, alkaloids, gums, glycosides, terpenoids, and steroids. This plant has antioxidant compounds, and the total content of phenolic, flavonoid, and tannin was measured at 38.7 mg GAE/g, 298.37 mg QE/g, and 43.9 mg GAE/g, respectively. In the 2,2-diphenyl-1-picrylhydrazyl (DPPH), H_2_O_2_, and superoxide scavenging tests, SC_50_ values were found to be 232.2, 131.67, and 192.12 *μ*g/mL, respectively. In the FeCl_3_ reducing assay, the RC_50_ was 79.71 *μ*g/mL. The *n-*hexane fraction showed a good antidiarrheal effect, while defecation inhibition was 54.42% and 66.29% at 250 and 500 mg/kg doses, respectively. Both *n-*hexane and ethyl acetate fractions exhibited a good antihyperglycemic effect in the oral glucose tolerance test in the experimental mice. In *α*-glucosidase inhibition, the IC_50_ value was 25.043 mg/mL. This extract did not show any promising responses in the diuretic, analgesic, and anthelmintic tests. From gas chromatography–mass spectrometry (GC-MS) investigation, we have found 25 compounds that are displayed in this extricate. After conducting absorption, distribution, metabolism, excretion, and toxicity (ADMET) and molecular coupling analysis, we finally selected four compounds which may be further tested in order to isolate newer antidiarrheal and antihyperglycemic compounds from this plant in the future.

## 1. Introduction

The use of medicinal plants for healing dates back to the inception of humanity. Recent years have seen a significant emphasis on exploring the potential health benefits offered by natural compounds that possess biological activities. Specific plants are acknowledged as notable reservoirs of nutrients and are consequently promoted for their therapeutic attributes. Illustrative examples of such plants include ginger, green tea, and walnuts. Furthermore, derivatives of select plants are identified as valuable repositories of active ingredients used in the formulation of aspirin and toothpaste [1]. The term “medicinal plants” denotes botanical species containing compounds or substances with therapeutic attributes that make them beneficial for application in traditional or contemporary medical practices. These plants are recognized as invaluable repositories of compounds with potential applications in pharmaceutical development and synthesis. Moreover, its historical significance extends to a pivotal role in the shaping of diverse human cultures worldwide [2]. The vast diversity of medicinal plants, estimated at around half a million globally, presents substantial untapped potential for future research. Many of these plant species remain inadequately examined for their medical properties. Investigating these underexplored botanical resources holds promise for addressing current and emerging health conditions.

The genus *Artocarpus* from the Moraceae family is a group of tall, woody plants. Many plants in this group are already famous for their various therapeutic importance. *Artocarpus chama* is such a plant from this genus that is actually native to some Asian countries like China, Malaysia, Bangladesh, Bhutan, India, Laos, Myanmar, and Thailand. This plant is known as Chapalish in Bangladesh and thrives in mountainous forests that extend from Chattogram, Chattogram Hill Tracts, Sylhet Division, and Madhupur forests in Tangail District. It is a sizable deciduous tree, known for its buttressed base, milky latex, and a height that reaches 30 m. The *Artocarpus* genus is rich in phenolic compounds, including flavonoids, stilbenoids, arylbenzofurons, and the lectin jacalin. Extracts and metabolites from different parts of the *Artocarpus* plant, such as leaves, bark, stems, and fruits, are recognized for their diverse and beneficial bioactive compounds [3]. Extracts of aerial and underground plant parts have been applied in traditional medicine for the treatment of diarrhea, diabetes, malarial fever, tapeworm infection, and other diseases. The other uses include wound healing; antisyphilitic; expectorant properties; and also used for the treatment of anemia, asthma, and dermatitis. Barks, leaves, seeds, and roots are used in diarrhea, skin diseases, asthma, ulcers, etc. [4]. Taking into account the wealth of bioactive compounds and the varied applications of this plant in traditional medicine, our aim was to conduct some pharmacological activities and in silico analysis of its leaves. The goal is to establish a scientific basis for its traditional usage.

## 2. Materials and Method

### 2.1. Plant Collection and Identification

**Figure 1:**
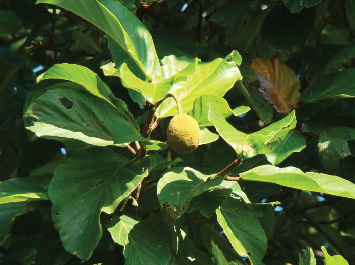
*Artocarpus chama* leaves and fruits.

In the present investigation, mature leaves of *A. chama* (Figure 1) were collected in January 2023 from Chittagong, Bangladesh, and later, dried plant samples were identified by specialists at the Bangladesh National Herbarium in Mirpur, Dhaka. A voucher specimen (Voucher Specimen No. DACB 85928) has been archived for future reference. The collected leaves were separated from all unwanted adulterants and dried in shade for 60 days. The dried leaves were then pulverized to a fine powder using a grinding machine and stored in airtight containers at room temperature.

### 2.2. Preparation of the Extract

Seven hundred grams of *A. chama* powder was placed in clean, flat-bottomed glass containers and immersed in 2000 mL of 96% ethanol for a duration of 14 days. The resulting filtrate, called the ethanol extract, was then concentrated using a rotary evaporator and then dried to finally obtain a 21.3 g gummy extract (yield = 3.04%).

#### 2.2.1. Fractionation

The ethanolic extract of *A. chama* was further separated into three fractions on the basis of their polarity. The crude extracts were then fractionated using *n*-hexane, ethyl acetate, and water resulting in the isolation of 10.7, 5.8, and 0.5 g of extract for the fractions, respectively. The yield of *n*-hexane, ethyl acetate, and water was 1.53%, 0.83%, and 0.07% of the extract for the fractions, respectively.

### 2.3. Chemicals and Reagents

The analytical experiments in the laboratory used high-quality reagents sourced from reputable suppliers. Acetic acid was obtained from the Merck Group in Darmstadt, Germany, while formalin, Folin–Ciocalteu (FC) reagent, and hydrogen peroxide (H_2_O_2_) were purchased from the same supplier. 2,2-Diphenyl-1-picrylhydrazyl (DPPH), gallic acid, FeCl_3_, nitrogen blue tetrazolium (NBT), phenazine methosulfate (PMS), and nicotine adenine dinucleotide (NADH) were purchased from Sigma–Aldrich Ltd. in Saint Louis, United States. Sodium carbonate (Na_2_CO_3_), sodium nitrite (NaNO_2_), disodium hydrogen phosphate dihydrate (Na_2_HPO_4_∙2H_2_O), sodium dihydrogen phosphate dihydrate (NaH_2_PO_4_∙2H_2_O), and aluminum chloride (AlCl_3_) were purchased from Loba Chemie Pvt. Ltd in Mumbai, India. Sodium hydroxide (NaOH) was also obtained from Loba Chemie Pvt. Ltd. All standard drugs used in the biological experiments were purchased from Square Pharmaceuticals, Bangladesh.

### 2.4. Animals

For the biological experiment, Swiss albino mice at the age of 6–7 weeks with body weights (bws) of 25–30 g were purchased from the animal house of Jahangirnagar University. These mice were kept in the Pharmacy Discipline Animal House, Khulna University, Bangladesh. The mice were housed under standard environmental conditions, provided with conventional laboratory food and tap water, and subjected to a natural day–night cycle. All experiments were carried out in a controlled and quiet environment, adhering to the ethical guidelines for animal handling established by the Animal Ethics Committee of Khulna University (KUAEC-2023-05-10).

### 2.5. Phytochemical Test

The *A. chama* extract was analyzed to ascertain the presence of various compounds such as reducing sugars, polyphenols, flavonoids, tannins, glycosides, steroids, terpenoids, and alkaloids. The identification of different phytoconstituents was carried out using the methodology outlined by Karmakar, Paul, and Bokshi [5].

### 2.6. In Vitro Antioxidant Tests

#### 2.6.1. Qualitative Antioxidant Test

The experiment utilized precoated silicon gel TLC plates, which were subsequently subjected to development in solvent systems categorized as polar, medium polar, and nonpolar. An appropriately diluted stock solution of the plant extract was applied to the TLC plates, followed by the application of a 0.02% DPPH solution in ethanol. Any hydrogen-donating molecules present in the extract were capable of reacting and decolorizing the DPPH [6].

#### 2.6.2. Quantitative Antioxidant Test

##### 2.6.2.1. Determination of Different Secondary Metabolite Content

*Determination of the total phenolic content (TPC).*

The determination of the TPC in the extract was carried out using the FC reagent, with analytical grade gallic acid serving as the standard. The TPC was quantified in milligrams of gallic acid equivalent per gram of dried extract using a gallic acid calibration curve [7].

*Determination of the total flavonoid content (TFC).*

The TFC of the extract was determined using AlCl_3_ colorimetric assay. Quercetin was used as the standard. Finally, the TFC was expressed as milligrams of quercetin equivalent per gram of dried extract using the quercetin calibration curve [8].

*Determination of the total tannin content (TTC).*

The TTC of the extract was determined using the FC reagents. Gallic acid was used as a standard. Finally, TTC was expressed as milligrams of equivalent gallic acid per gram of dried extract using the gallic acid calibration curve of gallic acid [7].

##### 2.6.2.2. Free Radical Scavenging Assay

*DPPH free radical scavenging assay.*

The evaluation of the DPPH free radical scavenging property of *A. chama* was carried out following the methodology outlined by Naznin et al. [9]. Various concentrations of plant extract solutions (1–4096 *μ*g/mL) were meticulously prepared, and the DPPH solution (using methanol as the solvent) was subsequently introduced into each solution. Absorbance measurements were recorded at 517 nm. The antioxidant activity was quantified in terms of IC_50_, which represents the concentration (micrograms per milliliter) of the plant sample required to scavenge 50% of DPPH free radicals. The SC_50_ value was determined by analyzing the curve depicting the logarithm of concentration versus percent scavenge.

*H_2_O_2_ scavenging assay.*

The H_2_O_2_ scavenging assay was carried out following the protocols outlined by Agrawal et al. with minor adjustments [10]. In this assay, solutions of *A. chama* extract were meticulously prepared at various concentrations (6.25–800 *μ*g/mL), and H_2_O_2_ (40 mM) was subsequently introduced to each solution. The absorbance at 230 nm was measured to assess the H_2_O_2_ scavenging effect of both the sample extract and standard compounds, and those were expressed as SC_50_.

*Superoxide radical scavenging assay.*

In the assessment of superoxide radical scavenging activity, the superoxide anion (O^2−^) generated from the PMS–NADH coupling reaction is responsible for reducing NBT to a purple form, making NBT a suitable indicator for quantifying the concentration of superoxide anion. The superoxide radical scavenging assay was conducted following the methodology outlined by Lin and Li [11]. In this experiment, *A. chama* extracts at various concentrations (6.25–1600 *μ*g/mL) were tested alongside NBT (312 *μ*M), NADH (936 *μ*M), and PMS (120 *μ*M). The efficacy of superoxide scavenging by plant extract and standard compounds was determined by analyzing the logarithmic concentration curve against the percent inhibition.

##### 2.6.2.3. Reducing Power Assay

The ferric reduction capacity of the plant extract was evaluated following the methodology outlined by Lin and Li [11] with minor adjustments. To perform the assay, varying concentrations of the sample solution (12.5–800 *μ*g/mL) were combined with 0.2 M phosphate buffer (2.5 mL) and 1% potassium ferricyanide (2.5 mL) and then subjected to incubation at 50°C. Subsequently, 10% trichloroacetic acid (TCA) (2.5 mL) was introduced, followed by centrifugation. The resulting solution was mixed with distilled water (2.5 mL) and ferric chloride (0.1%) (0.5 mL). The absorbance of the solution was measured at 700 nm.

### 2.7. Evaluation of Antidiarrheal Activity

An antidiarrheal test was performed on mice using castor oil–induced diarrhea outlined by Shoba and Thomas [12]. Castor oil is known to improve intestinal motility and decrease fluid absorption. The positive control group received loperamide at a dose of 3 mg/kg, while the test groups received sample extract at doses of 250 and 500 mg/kg bw. After a 30-min interval, 0.5 mL of castor oil was administered orally to induce diarrhea. Each mouse was then placed in an individual cage with blotting paper, and the latency period and stool count were recorded over a 4-h period.

### 2.8. Evaluation of Antihyperglycemic Activity

#### 2.8.1. Oral Glucose Tolerance Test (OGTT)

The oral OGTT is a widely recognized method used to evaluate the rate at which exogenous glucose is eliminated from the bloodstream. In this study, oral glucose tolerance and antihyperglycemic properties of a plant extract were evaluated following the protocol outlined by Lartey et al. [13]. Glibenclamide was administered at a dose of 5 mg/kg as standard drug. Subsequently, the glucose solution was administered orally at a rate of 2 g/kg bw to each mouse and blood glucose levels (measured in millimoles per liter) were evaluated at 0-, 30-, 60-, 90-, 120-, and 150-min intervals using a glucometer (Bionime GS100, Switzerland).

#### 2.8.2. Evaluation of *α*-Glucosidase Enzyme Inhibitory Activity

The inhibitory activity of *A. chama* extract on the alpha-glucosidase enzyme was evaluated following a method described by Telagari and Hullatti [14]. Acarbose served as the positive control in the study. Various concentrations of *A. chama* extract and acarbose were prepared. Subsequently, a mixture of potassium phosphate buffer, *α*-glucosidase enzyme, and p-nitrophenyl glucopyranoside was combined with extract and standard. The reaction was stopped by the addition of a sodium carbonate solution. Absorbance readings were then taken at 405 nm using a Thermo Scientific Multiskan GO spectrophotometer. The inhibitory activity of *α*-glucosidase was determined by plotting the logarithmic concentration against the percentage of inhibition and was quantified as IC_50_, representing the concentration of the sample required to inhibit 50% of the enzyme.

### 2.9. Evaluation of Diuretic Activity

The diuretic effect of fractionated extracts of *A. chama* was assessed following the methodology outlined by Karmakar et al. [15]. For comparison, a standard drug, furosemide at a dose of 5 mg/kg, was used. The fractionated extracts of *A. chama* were administered at doses of 250 and 500 mg/kg bw. The concentrations of furosemide and the fractionated extracts of *A. chama* extracts fractionated were adjusted to ensure that each animal received 2 mL of solution per administration. Diuretic activity was determined using the following equations.

 $$\begin{array}{c} \text{Urinary excretion}=\left[\text{total urinary output}\left(v_{\text{o}}\right)/\text{total liquid administered}\left(v_{\text{i}}\right)\right]\times 100 \end{array}$$

The diuretic effect was evaluated by dividing the urinary excretion of the test group by that of the control group, and subsequently, the diuretic activity was calculated by dividing the diuretic effect of the test group by that of the control group.

### 2.10. Evaluation of Analgesic Activity

The peripheral analgesic potential of *A. chama* extract was assessed by the acetic acid–induced writhing method [16]. Diclofenac Na, administered at a standard dose of 25 mg/kg bw, served as the reference drug. Following oral administration of the plant sample at doses of 250 and 500 mg/kg bw, pain induction was initiated 30 min later using 0.7% acetic acid administered intraperitoneally. Pain responses, characterized by abdominal constriction, trunk twisting, and hind leg extension, were observed. The counting of writhing episodes occurred after a 15-min interval, with observations recorded over a 5-min duration.

### 2.11. Evaluation of Anthelmintic Activity

The anthelmintic effect of different fractions of *A. chama* extract was determined in *Paramphistomum cervi* helminths according to the method described by Dasi, Dey, and. Ghosh [17]. From a local abattoir, we have collected *P. cervi* nematodes from the intestines of freshly slaughtered cattle. Those nematodes were frequently washed with 0.9% NaCl solution. Different fractions of *A. chama* extract were prepared at 25, 50, 100, and 200 mg/mL. Along with these, albendazole was prepared at 15 mg/mL. Ten milliliters of each prepared concentration was placed in separate Petri dishes, while each contains five helminths. Those were kept to count the paralysis time (normally, no movement, but move after shaking) and death time (no movement after vigorous shaking).

### 2.12. Gas Chromatography–Mass Spectrometry (GC-MS) Analysis

This analysis was carried out with a Clarus 690 gas chromatograph (PerkinElmer, California, United States) using a column (Elite-35, 30-m length, 0.25-mm diameter, and 0.25-*μ*m thickness of film), and it was equipped with a Clarus SQ 8C mass spectrophotometer (PerkinElmer, California, United States). A 1-*μ*L sample was injected (split mode), and pure helium (99.999%) was used as a carrier gas at a constant flow rate (1 mL/min) of 40-min run time. The sample was analyzed in the electron ionization (EI) mode at high energy (70 eV). Although the inlet temperature was constant at 280°C, the column oven temperature was set at 60°C (for 0 min), increased at 5°C per minute to 240°C, and held for 4 min [18].

### 2.13. In Silico Analysis

#### 2.13.1. Absorption, Distribution, Metabolism, Excretion, and Toxicity (ADMET) Analysis

From the results of GC-MS analysis, we have found that there are 25 compounds present in this extract of *A. chama.* To determine the safety analysis of these ligands, ADMET analysis was performed considering the famous Lipinski rule of five. We have collected the pharmacokinetic parameters of ligands from the SwissADME and pkCSM site [19].

#### 2.13.2. Molecular Docking

From the above pharmacological tests, we have observed that fractions of *A. chama* only showed good responses in antidiarrheal and antihyperglycemic tests. So, we now conduct molecular docking on these two tests. At first, the protein models Protein Data Bank (PDB) ID 4U14 (*μ*-opioid receptor) and 5NN8 (*α*-glucosidase enzyme) were downloaded from the PDB (https://www.rcsb.org/). Those proteins were further processed by Discovery Studio 2020 in order to remove all attached ligands and water molecules. Then, compounds that did not violate the Lipinski rule of five and also showed no hepatotoxicity and AMES toxicity were selected and their 3D structures were downloaded from the PubChem site (https://pubchem.ncbi.nlm.nih.gov/). The 19 downloaded ligands were (3E,7E)-4,8,12-trimethyltrideca-1,3,7,11-tetraene (CID: 6443227); (R)-(-)-4-methylhexanoic acid (CID: 12600623); 1H-pyrrole-2,5-dione, 3-ethyl-4-methyl (CID: 29995); 1-propanone, 2-bromo-1-phenyl (CID: 6931748); 1-[(trimethylsilyl)oxy]propan-2-ol (CID: 23105108); 1r,2c,3t,4t-tetramethyl-cyclohexane (CID: 94277); 2H-pyran, 2-(7-dodecynyloxy)tetrahydro (CID: 86051); bis(2-ethylhexyl) phthalate (CID: 8343); cyclohexanepropanoic acid, 2-oxo-, methyl ester (CID: 112036); ethyl 13-methyl-tetradecanoate (CID: 71380066); heptacosanoic acid, 25-methyl-, methyl ester (CID: 554101); methyl 8,11,14-heptadecatrienoate (CID: 91697551); neophytadiene (CID: 10446); n-hexadecanoic acid (CID: 985); phthalic acid, di(trans-dec-3-enyl) ester (CID: 91694627); phytyl decanoate (CID: 140471960); tetradecanoic acid, 10,13-dimethyl-, methyl ester (CID: 554145); tricosanal (CID: 155761); and tridecanoic acid, 12-methyl-, methyl ester (CID: 21204). Standard drugs, acarbose (CID: 9811704) and loperamide (CID: 3955), were also downloaded from the same site. The energy minimization of those ligands was processed using PyRx. We have decided to conduct blind coupling to ensure that all protein sites become available for binding where the grid dimensions for 4U14 and 5NN8 were *x* : *y* : *z* = 59.2274 : 61.7882 : 92.9343 and 82.0615:80.9558:87.9160, respectively [20].

### 2.14. Statistical Analysis

The experimental data presented here are expressed as mean = (number ± standard error of the mean (SEM)). Using Statistical Package for the Social Sciences (version 25), we had analyzed the data using one-way ANOVA followed by Tukey as post hoc test. The statistical significance in all cases was considered *p* < 0.05. The graphic images presented in this manuscript were prepared using GraphPad Prism software (version 21) [8].

## 3. Results

### 3.1. Phytochemical Test

Examination of the extract of *A. chama* revealed the existence of various important phytochemical groups such as reducing sugars, tannins, flavonoids, alkaloids, gums, glycosides, terpenoids, and steroids.

### 3.2. Antioxidant Tests

The plates were examined using a UV detector at both short (254 nm) and long (360 nm) wavelengths. The UV detector revealed numerous fluorescent and colored positive components at shorter and longer wavelengths, respectively. TLC was conducted using nonpolar, medium polar, and polar solvent systems. Following the application of DPPH to the TLC plate, a yellow color was observed against a purple background, indicating the presence of antioxidant components in the extract of *A. chama*. From the quantification of different secondary metabolite contents, we have estimated their values (Table 1). And by obtaining the calibration curves for the relevant standards, as well as for *A. chama* extract, we have determined the SC_50_ and RC_50_ values for this extract (Table 1).

### 3.3. Evaluation of Antidiarrheal Activity

In castor oil–induced diarrheal mice, the *n*-hexane fraction of *A. chama* extract at a dose of 250 and 500 mg/kg bw significantly reduced the total number of feces, as well as delayed the onset of diarrhea in a dose-dependent manner. Percentage inhibition of defecation for *n*-hexane fraction *A. chama* at doses 250 and 500 mg/kg bw was 54.42% and 66.29%, respectively. The mean latent period for *A. chama* at doses 250 and 500 mg/kg bw was 129.5 and 152.1 min, respectively. On the contrary, the ethyl acetate fraction of the same plant did not show any notable antidiarrheal activity (Table 2).

### 3.4. Evaluation of Antihyperglycemic Activity

**Figure 2:**
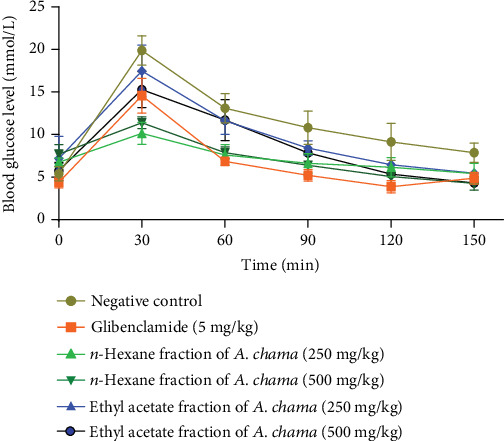
Comparison of blood glucose level in OGTT at different times for negative control, glibenclamide, and different fractions of *A. chama* extract.

**Figure 3:**
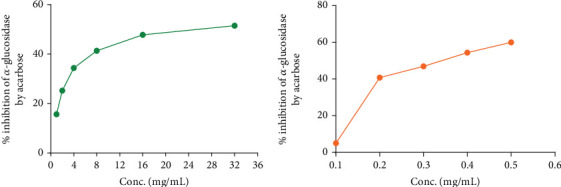
Graphical presentation of *α*-glucosidase enzyme inhibitory activity of (a) *A. chama* extract and (b) acarbose.

In the OGTT test, we have seen that both fractions of *A. chama* extract at both doses reduced the elevated blood glucose level over time in a dose-dependent manner (Figure 2). It signifies the antihyperglycemic effect. And later, the IC_50_ for the crude extract and acarbose of *A. chama* was 25.043 and 0.37 mg/mL, respectively (Figure 3).

### 3.5. Evaluation of Diuretic Activity

**Figure 4:**
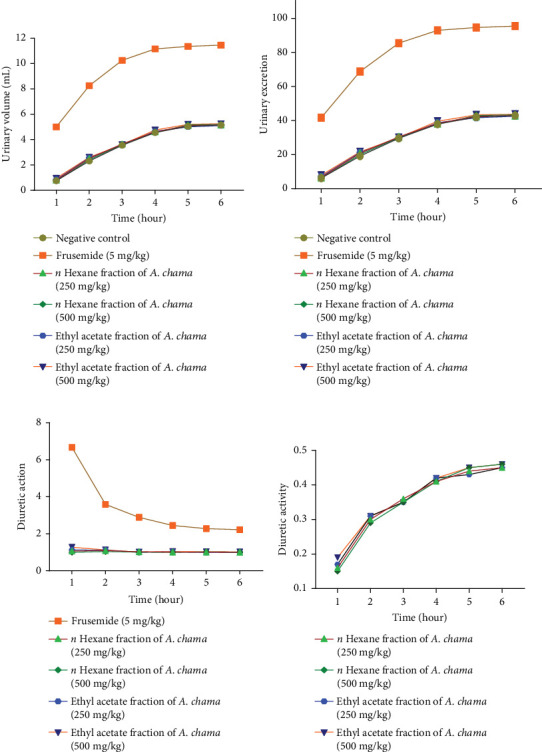
(a) Urinary volume, (b) urinary excretion, (c) urinary action, and (d) urinary activity of different fractions of *A. chama* extract.

For both fractions, even at both doses, there was no significant diuretic effect. Figures 4(a), 4(b), 4(c), and 4(d) show urinary volume, urinary excretion, diuretic action, and diuretic activity, respectively.  Table 3 exhibits the different electrolyte concentrations and their related data due to the effect of both fractions of *A. chama* in mice.

### 3.6. Evaluation of Analgesic Activity

The results showed that none of the fractions have analgesic activity compared to standard diclofenac sodium. Inhibition of writing for the *n*-hexane fraction at 250 and 500 mg/kg doses was found to be 8.38% and 12.42%, respectively, while for the ethyl acetate fraction, it was 9.63% and 12.73%, respectively. The standard drug diclofenac Na inhibited writhing 75.46% at a dose of 25 mg/kg bw (Table 4).

### 3.7. Evaluation of Anthelmintic Activity

None of the fractions of *A. chama* showed any anthelmintic activity. Despite trying with four different concentrations, both fractions remain ineffective to paralyze or kill the helminth. Here, the standard (albendazole) showed approximately 6.83 min of paralysis and 8.33 min of death at the dose of 15 mg/mL against the parasite.

### 3.8. GC-MS Analysis of *A. chama* Extract

**Figure 5:**
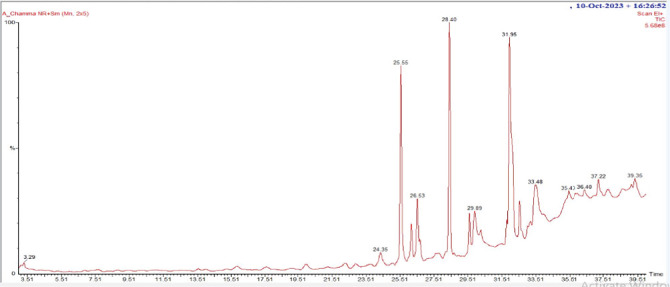
GC-MS chromatogram of the extract of *A. chama*.

**Figure 6:**
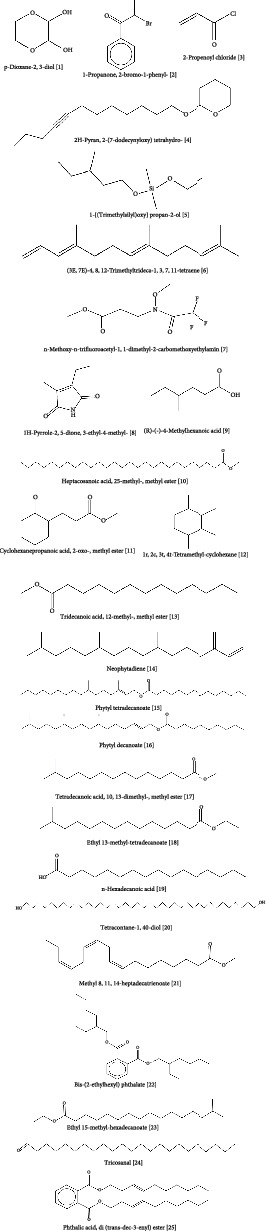
Chemical structures of 25 compounds in *A. chama* extract from GC-MS analysis.

In the GC-MS analysis, 26 compounds were identified from *A chama*.  Figure 5 represents the distinct GC-MS chromatogram. The bioactive compounds identified from *A chama* were represented by their retention time (RT), molecular weight, and peak area (percentage) in Table 5. The chemical structures of these 26 compounds are depicted in Figure 6.

### 3.9. In Silico Analysis

From ADMET analysis, we have listed the pharmacokinetic parameters of 25 compounds in Table 6. From this table, we have found that two compounds (phytyl tetradecanoate and tetracontane-1,40-diol) violate the Lipinski rule of five and four compounds show hepatotoxicity and AMES toxicity (Table 6).

In molecular coupling, we have performed coupling of 18 compounds against the 4U14 and 5NN8 proteins. Only 1-[(trimethylsilyl) oxy] propan-2-ol could not be correlated with these proteins by this software. Therefore, we excluded this compound from docking. The docking scores of the remaining 18 compounds along with two standard drugs are presented in Table 7.

**Figure 7:**
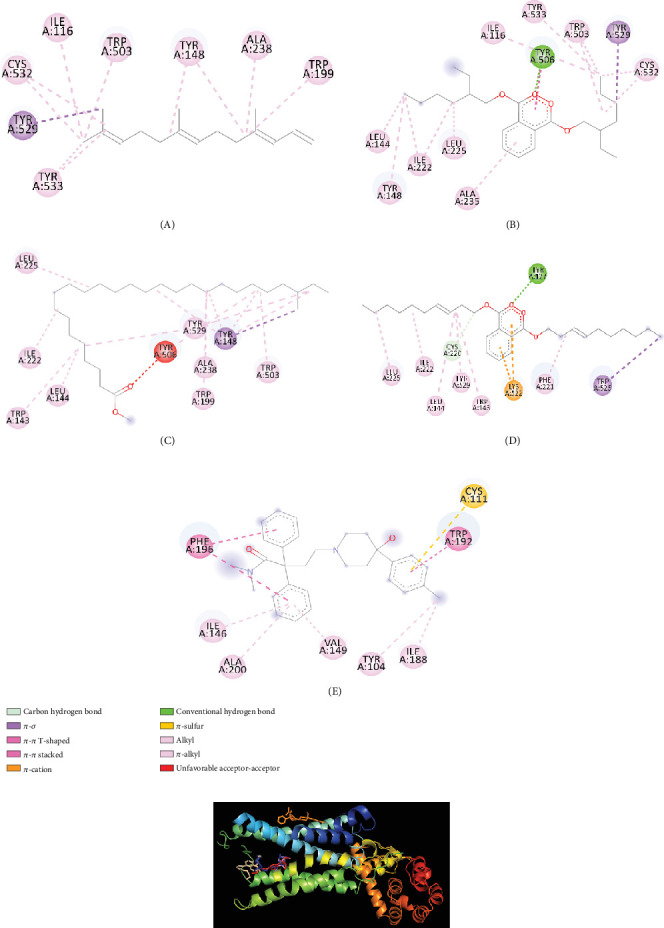
(a) 2D interactions of amino acids of the 4U14 protein with (A) (3E,7E)-4,8,12-trimethyltrideca-1,3,7,11-tetraene; (B) bis(2-ethylhexyl) phthalate; (C) heptacosanoic acid, 25-methyl-, methyl ester; (D) phthalic acid, di(trans-dec-3-enyl) ester; and (E) loperamide. (b) Binding pocket of (3E,7E)-4,8,12-trimethyltrideca-1,3,7,11-tetraene (violet); bis(2-ethylhexyl) phthalate (red); heptacosanoic acid, 25-methyl-, methyl ester (blue); loperamide (orange); and phthalic acid, di(trans-dec-3-enyl) ester (wheat) with 4U14 protein.

After molecular coupling with the 4U14 protein, only four compounds named as (3E,7E)-4,8,12-trimethyltrideca-1,3,7,11-tetraene (**6**); bis (2-ethylhexyl) phthalate (**22**); phthalic acid, di(trans-dec-3-enyl) ester (**25**); and heptacosanoic acid, 25-methyl-, methyl ester (**10**) showed binding affinities greater than 7 kcal/mol. The interactions of these compounds with the 4U14 protein along with their interacting amino acids are presented in Table 8. The 2D and 3D interactions are presented in Figures 7(a) and 7(b), respectively.

**Figure 8:**
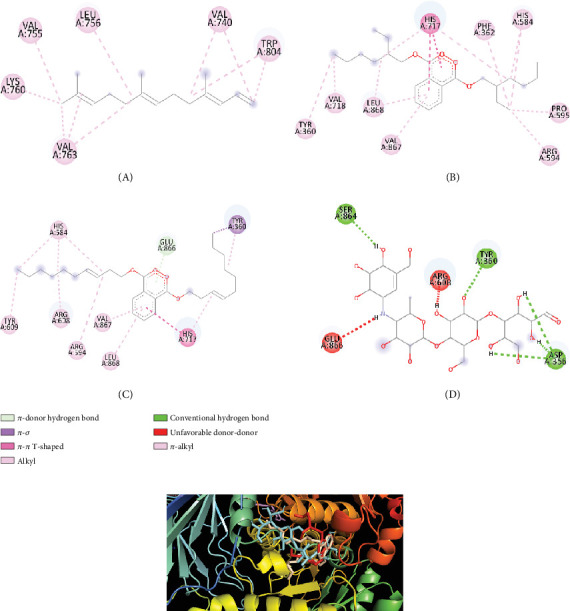
(a) 2D interactions of amino acids of the 5NN8 protein with (A) (3E,7E)-4,8,12-trimethyltrideca-1,3,7,11-tetraene; (B) bis(2-ethylhexyl) phthalate; (C) phthalic acid, di(trans-dec-3-enyl) ester; and (D) acarbose. (b) Binding pocket of (3E,7E)-4,8,12-trimethyltrideca-1,3,7,11-tetraene (violet); acarbose (cyans); bis(2-ethylhexyl) phthalate (red); and phthalic acid, di(trans-dec-3-enyl) ester (wheat) with the 5NN8 protein.

And in coordination with the 5NN8 protein, only three compounds named (3E,7E)-4,8,12-trimethyltrideca-1,3,7,11-tetraene; bis (2-ethylhexyl) phthalate; and phthalic acid, di(trans-dec-3-enyl) ester showed binding affinities greater than 6 kcal/mol. Interactions of these compounds with 5NN8 protein along with their interacting amino acids are presented in Table 9. 2D and 3D interactions are presented in Figures 8(a) and 8(b), respectively.

## 4. Discussion

Plants in the natural environment contain a wide range of phytochemicals that can be categorized into primary and secondary metabolites on the basis of their chemical composition and biosynthetic origin. Secondary metabolites, in particular, play a crucial role in manifesting various biological functions. These phytochemicals demonstrate a range of pharmacological properties that are advantageous both to the plant and to humans. Throughout history, humans have sought remedies for ailments by utilizing the medicinal properties of these plants since ancient times [21]. The phytochemical tests carried out on *A. chama* extract revealed the presence of several important constituents that could be responsible for its traditional medicinal uses. The phytochemical groups that have been found are carbohydrate, alkaloid, phenolic compounds, flavonoids, tannins, glycoside, protein and amino acids, saponin, gum, and acidic compounds.

Antioxidants are molecules that counteract the effects of harmful compounds known as free radicals, which can cause cell damage, food spoilage, and degradation of various materials such as rubber, gasoline, and lubricating oils. Free radicals are formed when oxygen interacts with specific molecules, resulting in atoms or groups of atoms with an unpaired number of electrons. These free radicals are generated under certain environmental conditions and as part of normal cellular processes in the body. The body uses an antioxidant defense system to prevent free radical damage caused by free radicals [22]. Antioxidants interrupt the chain reaction initiated by free radicals. Although some antioxidants are themselves free radicals that donate electrons to stabilize and neutralize harmful free radicals, others target the molecules that give rise to free radicals, eliminating them before they can trigger oxidative damage. The DPPH radical is a stable radical commonly used that accepts an electron or proton from antioxidant compounds, resulting in conversion to a yellowish hue [23]. H_2_O_2_ serves as a potent oxidative agent that produces hydroxyl radicals (OH) within aqueous environments. On the contrary, the superoxide radical poses a threat to cell constituents, acting as a precursor to reactive oxygen species (ROS) that inflict damage on tissues and cells, leading to the onset of diverse diseases [24]. Antioxidants play a crucial role in counteracting the deleterious effects of ROS [25].

From Table 1, we can say that this plant showed little antioxidant activity. Preliminary phytochemical studies of *A. chama* showed the presence of flavonoids and other phenolic compounds; therefore, suppression of released H_2_O_2_ may be attributed to direct H_2_O_2_ scavenging. The antioxidant reduces the adverse effect of wounds by removing inflammation products. They counteract excess proteases and ROS often formed by neutrophil accumulation in the injured site and protect protease inhibitors from oxidative damage.

Castor oil contains approximately 90% ricinolate, an active metabolite that induces diarrhea by reducing the permeability of sodium and chloride ions in the intestine and stimulating the release of prostaglandins. The antidiarrheal properties of medicinal plants are attributed to compounds such as tannins, alkaloids, flavonoids, steroids, terpenoids, and reducing sugars [26]. In this study, the plant extract demonstrated antidiarrheal effects comparable to loperamide, a commonly used antidiarrheal drug. The extract antagonized diarrhea induced by castor oil and prostaglandins, possibly through its antimotility and antisecretory properties. Tannins and alkaloids present in plant extract are known to improve intestinal mucosal resistance and reduce secretion, thus inhibiting castor oil–induced diarrhea. These phytochemical groups are predominantly found in the leaves of the plant, suggesting that the presence of tannins and alkaloids may contribute to the observed antidiarrheal activity.

Diabetes mellitus is a multifaceted condition marked by significant changes in the metabolism of carbohydrates, proteins, and fats. It represents a progressive disorder of glucose metabolism that ultimately results in vascular changes at both micro- and macrolevels, leading to challenging secondary complications. It is caused by inadequate insulin synthesis or inactivation of secreted insulin [27]. Various natural compounds derived from plants, including glycosides, alkaloids, terpenoids, carotenoids, and flavonoids, have demonstrated efficacy as antidiabetic agents in both preclinical and clinical investigations [28]. In our OGTT experiment, we found that both fractions showed a reduction in blood glucose in experimental mice. In comparison, the *n*-hexane fraction exhibited more antihyperglycemic activity than the ethyl acetate fraction of *A. chama*. An approach used in the treatment of diabetes mellitus is the inhibition of carbohydrate digesting enzymes, specifically alpha-glucosidase, in the gastrointestinal tract. This action is aimed at reducing glucose absorption after meals, thereby lowering postprandial glucose levels [29]. From the Figure 3, we have found that the inhibitory capacity of *A. chama* extract on the alpha-glucosidase enzyme is too little to mention. So, we may conclude that *A. chama* reduced blood glucose level other than inhibition of *α*-glucosidase enzyme mechanism.

Diuretics are pharmacological agents that improve urine flow and sodium excretion, commonly used to regulate body fluid volume and composition in various clinical conditions such as hypertension, heart failure, renal failure, nephrotic syndrome, and cirrhosis. In addition to influencing sodium excretion, diuretics can also affect the renal handling of other electrolytes (e.g., potassium, hydrogen ions, calcium, and magnesium), anions (e.g., chloride, bicarbonate, and dihydrogen phosphate), and uric acid [30]. Within the medical field, diuretics are used for the management of heart failure, liver cirrhosis, hypertension, influenza, water intoxication, and specific renal disorders. In particular, certain diuretics, such as thiazides and loop diuretics, exhibit antihypertensive effects that are distinct from their diuretic properties [31]. The study served as a secondary purpose of evaluating the use of these leaves to evaluate the diuretic effect. No increase in urine volume was found after 6 h in either fraction of *A. chama*, indicating the absence of presence of diuretic active compound in the test sample.

The acetic acid–induced writhing test is a widely accepted method to assess the analgesic properties of medicinal agents. Pain experienced in this test is triggered by the release of arachidonic acid from tissue phospholipids through the COX enzyme, leading to the production of prostaglandins such as PGE_2_ and PGE_2*α*_, as well as an increase in lipoxygenase products in the peritoneal fluid. These prostaglandins and lipoxygenase products contribute to swelling and pain by increasing capillary permeability and releasing endogenous substances that activate pain receptors. Nonsteroidal anti-inflammatory drugs (NSAIDs) work by inhibiting the COX enzyme in peripheral tissues, thereby affecting the transmission of pain signals from nociceptors [32]. The results of our study of the acetic acid–induced abdominal constriction assay did not show a notable decrease in the writhing reflex. These findings suggest that *A. chama* extracts do not possess potent analgesic compounds.

One of the most common parasitic infestations that cause morbidity and mortality in people and animals worldwide is the helminth infestation. It is one of the main factors contributing to the high rates of pneumonia, eosinophilia, anemia, and malnutrition. People who live in unclean environments, do not know enough about sanitation, or experience damp weather are more likely to contract helminthiasis. Helminthiasis also seriously affects livestock production. These parasitic worms secrete toxins and deprive host bodies of essential micronutrients while living in their stomachs. Consequently, the host experiences weight loss, reduced milk production, and malnutrition. Therefore, plant-derived new anthelmintic is always in great demand to scientists and dairy producers. Unfortunately, none of the extract fractions of *A. chama* showed any sort of anthelmintic effect on *P. cervi*.

After conducting the above biological tests, we decided to conduct a GC-MS analysis of the crude extract of *A. chama*. This is a robust technique to determine different chemical constituents from a mixture or crude extract. From our GC-MS analysis, we have found the existence of 25 compounds from the crude extract. According to the retention area, tetracontane-1,40-diol; tetradecanoic acid, 10,13-dimethyl-, methyl ester; and neophytadiene were found mainly in the extract and their percentage of retention area was 21.84, 13.18, and 12.37, respectively.

In the last decades, ADMET analysis has become a very important concern in determining the safety profile of any compound. Its complete comprehensive pharmacokinetic analysis of any compound mainly determines whether it will be safe or toxic for human use. Nowadays, there are many online tolls that are available that are helpful in predicting this behavior for a drug candidate. In our analysis, we have collected those data from the SwissADME and pkCSM site. We have mainly focused on the famous Lipinski rule of five. According to this rule, a compound must satisfy any four criteria of the following five: must have a molecular weight 500 Da, five hydrogen bond donors, 10 hydrogen bond acceptors, lipophilicity 5, and finally molar refractivity must be 40–130. From our analysis, two compounds out of 25 violate this rule. In next, more four compounds showed AMES or hepatotoxicity. For that reason, we may consider that the remaining 19 compounds may be safe for human use.

Afterwards, we conducted molecular docking, which is a very essential process in order to determine the binding interaction between the drug and other respective receptors (protein molecules). Molecular docking states that the activities of a compound increase with its affinity for proteins. Characterizing binding behavior is essential for the rational development of pharmaceuticals and for shedding light on fundamental biological processes. Only one compound named 1-[(trimethylsilyl)oxy]propan-2-ol could not be docked by PyRx software. The remaining 18 compounds were docked, and from the docking score, we have seen that four compounds named (3E,7E)-4,8,12-trimethyltrideca-1,3,7,11-tetraene; bis (2-ethylhexyl) phthalate; heptacosanoic acid, 25-methyl-, methyl ester; and phthalic acid, di(trans-dec-3-enyl) ester showed some binding affinities with receptor protein molecules. Most of the cases, pi-sigma, pi-pi-t-shaped, and pi-pi alkyl bonds were also visualized. So, finally we may suggest that only these four compounds could be processed for further research in isolating newer antidiarrheal and antihyperglycemic compounds.

## 5. Conclusions

This research was carried out on *A. chama* leaves in order to assess its pharmacological and antioxidant effects. From our result, we can conclude that this plant might be effective in antidiarrheal and antihyperglycemic purposes, as we have found promising responses in these two tests. However, in other tests such as analgesic, diuretic, and anthelmintic, the extract did show any notable effects. This plant also showed radical scavenging effects, as well as the possession of different antioxidative compounds. From the results of GC-MS analysis and after ADMET analysis and molecular docking, finally, we have selected four compounds which may be used in conducting further research in isolating newer and better lead molecules from this plant in the future.

## Figures and Tables

**Table 1:** Approximate SC_50_ values of different radical scavenging assays, RC_50_ in ferric reducing assay, and total content of secondary metabolites (phenolics, flavonoids, and tannins) of extract of *A. chama* leaves.

**Sample**	**TPC ** **(mg GAE/g)**	**TFC ** **(mg QE/g)**	**TTC ** **(mg GAE/g)**	**DRSA ** **(SC**_**50**_ ***μ*g/mL)**	**HPSA ** **(SC**_**50**_ ***μ*g/mL)**	**SRSA ** **(SC**_**50**_ ***μ*g/mL)**	**RPA ** **(RC**_**50**_ ***μ*g/mL)**
*A. chama* extract	38.702	298.366	43.909	232.18	131.667	192.12	79.71
Ascorbic acid	—	—	—	41.32	101.19	114.36	28.09

Abbreviations: DRSA, DPPH radical scavenging activity; HPSA, hydrogen peroxide scavenging activity; RPA, reducing power assay; SRSA, superoxide radical scavenging activity; TFC, total flavonoid content; TPC, total phenolic content; TTC, total tannin content.

**Table 2:** Representation of the effects of different fractions of *A. chama* extract on castor oil–induced diarrheal mice.

**Treatment groups**	**Dose (mg/kg)**	**Mean latent period (min)**	**No. of stool in 4 h**	**% inhibition of defecation**
Control 0.1% Tween-80 in water	—	38 ± 1.47^#□■^	17.8 ± 0.95^#□■○^	—
Standard (loperamide)	3 mg/kg	190.6 ± 1.47^∗^^□■○●^	2.4 ± 0.27^∗^^□■○●^	86.52
*n*-Hexane fraction of *A. chama*	250 mg/kg	129.5 ± 1.81^∗^^#■○●^	7.4 ± 0.34^∗^^#○●^	54.42
*n*-Hexane fraction of *A. chama*	500 mg/kg	152.1 ± 1.45^∗^^#□○●^	6 ± 0.29^∗^^#○●^	66.29
Ethyl acetate fraction of *A. chama*	250 mg/kg	38 ± 1.67^#□■^	17.6 ± 1.14^∗^^#□■^	1.12
Ethyl acetate fraction of *A. chama*	500 mg/kg	42.5 ± 2.2^#□■^	17.2 ± 1.39^#□■^	3.37

*Note:* Data are means of five replicates ± SEM (standard error of the mean).

⁣^∗^*p* < 0.05 versus negative control (Dunnett's *t* test).

^#^ *p* < 0.05 versus loperamide 3 mg/kg.

^□^ *p* < 0.05 versus *n*-hexane fraction of *A. chama* extract 250 mg/kg.

^■^ *p* < 0.05 versus ethyl acetate fraction of *A. chama* extract 500 mg/kg.

^○^ *p* < 0.05 versus ethyl acetate fraction of the *A. chama* extract 250 mg/kg.

^●^ *p* < 0.05 versus ethyl acetate fraction of *A. chama* extract 500 mg/kg (pair comparison by the post hoc Tukey test).

**(a):** 

**Treatment groups**	**Na** ^**+**^ ** (mEq/L)**	**K** ^**+**^ ** (mEq/L)**	**Cl** ^**−**^ ** (mEq/L)**	**Cumulative saluretic ** **(Na** ^**+**^ **+ Cl**^**−**^**)**	**Natriuretic ** **(Na** ^**+**^ **/K** ^**+**^ **)**	**Kaliuretic ** **(K** ^**+**^ **/Na+)**	**CAI (Cl** ^**−**^/**(Na**^**+**^ **+ K**^**+**^**))**
Negative control	24.77 ± 3.39	13.76 ± 0.87	29.17 ± 4.17	53.94	1.80	0.55	0.76
Furosemide (5 mg/kg)	72.22 ± 3.39	45.49 ± 1.74	106.25 ± 7.22	178.47	1.58	0.63	0.90
*n*-Hexane fraction of *A. chama* (250 mg/kg)	32.73 ± 5.87	11.35 ± 2.87	31.77 ± 5.87	64.5	2.79	0.36	0.71
*n*-Hexane fraction of *A. chama* (500 mg/kg)	33.59 ± 8.97	13.91 ± 1.97	30.56 ± 8.97	64.15	2.47	0.40	0.65
Ethyl acetate fraction of *A. chama* (250 mg/kg)	30.28 ± 3.39	14.20 ± 4.10	28.25 ± 6.39	58.53	2.12	0.47	0.63
Ethyl acetate fraction of *A. chama* (500 mg/kg)	33.81 ± 8.97	14.41 ± 3.97	29.89 ± 7.97	63.7	2.28	0.42	0.61

*Note:* Data are presented here mean ± standard error of the mean (SEM).

**(b):** 

**Treatment groups**	**Na** ^**+**^ ** index**	**K** ^**+**^ ** index**	**Cl** ^**−**^ ** index**	**Cumulative saluretic index**	**Natriuretic index**	**Kaliuretic index**	**CAI index**
Negative control	1	1	1	1	1.80	0.55	0.76
Furosemide (5 mg/kg)	2.20	2.80	3.33	2.765	1.58	0.63	0.90
*n*-Hexane fraction of *A. chama* (250 mg/kg)	1.09	1.03	1.07	1.08	2.79	0.36	0.71
*n*-Hexane fraction of *A. chama* (500 mg/kg)	1.03	1.05	1.03	1.03	2.47	0.40	0.65
Ethyl acetate fraction of *A. chama* (250 mg/kg)	1.08	1.02	1.07	1.075	2.12	0.47	0.63
Ethyl acetate fraction of *A. chama* (500 mg/kg)	1.02	1.07	1.10	1.06	2.28	0.42	0.61

Data are presented here mean ± standard error of the mean (SEM).

**Table 4:** Representation of the effects of different fractions of *A. chama* extracts on acetic acid induced in mice.

**Treatment group**	**Dose (mg/kg)**	**Mean writhing**	**% inhibition of writhing**
Control0.1% Tween-80 in water	—	32.20 ± 1.53^#●^	—
Standard (diclofenac Na)	25 mg/kg	7.9 ± 0.56^∗^^□■○●^	75.46
*n*-Hexane fraction of *A. chama*	250 mg/kg	29.50 ± 0.97^∗^^#□■○●^	8.38
*n*-Hexane fraction of *A. chama*	500 mg/kg	28.20 ± 1.14^#^	12.42
Ethyl acetate fraction of *A. chama*	250 mg/kg	29.1 ± 0.9^#^	9.63
Ethyl acetate fraction of *A. chama*	500 mg/kg	28.1 ± 1.13^∗^^#^	12.73

*Note:* Data are means of five replicates ± SEM (standard error of the mean).

⁣^∗^*p* < 0.05 versus negative control (Dunnett's *t* test).

^#^ *p* < 0.05 versus diclofenac Na 25 mg/kg.

^□^ *p* < 0.05 versus *n*-hexane fraction of *A. chama* extract 250 mg/kg.

^■^ *p* < 0.05 versus *n*-hexane fraction of *A. chama* extract 500 mg/kg.

^○^ *p* < 0.05 versus ethyl acetate fraction of *A. chama* extract 250 mg/kg.

^●^ *p* < 0.05 versus ethyl acetate fraction of the *A. chama* extract 500 mg/kg (pair comparison by post hoc Tukey test).

**Table 5:** Phytochemical composition of *A chama* from GC-MS analysis.

**Serial no.**	**Retention time (RT)**	**Name of the compounds**	**Molecular weight**	**% area**
1	3.29	p-Dioxane-2,3-diol	120	0.01
2	4.29	1-Propanone, 2-bromo-1-phenyl-	212	0.05
3	5.98	2-Propenoyl chloride	90	0.09
4	6.91	2H-Pyran, 2-(7-dodecynyloxy) tetrahydro-	266	0.16
5	7.66	1-[(Trimethylsilyl) oxy] propan-2-ol	148	0.57
6	8.80	(3E,7E)-4,8,12-Trimethyltrideca-1,3,7,11-tetraene	218	0.17
7	9.45	n-Methoxy-n-trifluoroacetyl-1,1-dimethyl-2-carbomethoxyethylamin	257	0.15
8	15.85	1H-Pyrrole-2,5-dione, 3-ethyl-4-methyl-	139	1.18
9	17.58	(R)-(-)-4-Methylhexanoic acid	130	0.75
10	19.98	Heptacosanoic acid, 25-methyl-, methyl ester	438	0.82
11	22.29	Cyclohexanepropanoic acid, 2-oxo-, methyl ester	184	1.20
12	22.89	1r,2c,3t,4t-Tetramethyl-cyclohexane	140	0.68
13	24.35	Tridecanoic acid, 12-methyl-, methyl ester	242	1.86
14	25.55	Neophytadiene	278	12.37
15	26.17	Phytyl tetradecanoate	506	2.14
16	26.53	Phytyl decanoate	450	4.67
17	28.40	Tetradecanoic acid, 10,13-dimethyl-, methyl ester	270	13.18
18	29.58	Ethyl 13-methyl-tetradecanoate	270	1.84
19	29.89	n-Hexadecanoic acid	256	3.76
20	31.95	Tetracontane-1,40-diol	594	21.84
21	32.54	Methyl 8,11,14-heptadecatrienoate	278	2.15
22	33.48	Bis(2-ethylhexyl) phthalate	390	10.07
23	36.40	Ethyl 15-methyl-hexadecanoate	298	0.80
24	37.22	Tricosanal	338	1.54
25	39.35	Phthalic acid, di(trans-dec-3-enyl) ester	442	2.08

*Note:*Sample compounds were identified compared with those of the National Institute of Standards and Technology (NIST) database.

**Table 6:** ADMET analysis of ligands in *A. chama* extract.

**Serial no.**	**Name of the ligands**	**MW**	**NHA**	**NHD**	**MR**	**Log ** **p**	**HT**	**AT**	**NLV**	**DL**
1	p-Dioxane-2,3-diol	120.10	4	2	23.72	−1.33	No	**Yes**	0	Yes
2	1-Propanone, 2-bromo-1-phenyl-	213.07	1	0	49.31	2.6527	No	No	0	Yes
3	2-Propenoyl chloride	90.51	1	0	21.06	0.9378	No	**Yes**	0	Yes
4	2H-Pyran, 2-(7-dodecynyloxy) tetrahydro-	266.42	2	0	82.05	4.6737	No	No	0	Yes
5	1-[(Trimethylsilyl) oxy] propan-2-ol	148.28	2	1	41.07	1.2187	No	No	0	Yes
6	(3E,7E)-4,8,12-Trimethyltrideca-1,3,7,11-tetraene	218.38	0	0	77.13	5.5916	No	No	1	Yes
7	n-Methoxy-n-trifluoroacetyl-1,1-dimethyl-2-carbomethoxyethylamin	243.18	7	0	46.26	0.8903	No	**Yes**	0	Yes
8	1H-Pyrrole-2,5-dione, 3-ethyl-4-methyl-	139.15	2	1	40.29	0.3693	No	No	0	Yes
9	(R)-(-)-4-Methylhexanoic acid	130.18	2	1	37.53	1.8973	No	No	1	Yes
10	Heptacosanoic acid, 25-methyl-, methyl ester	438.77	2	0	142.80	10.1778	No	No	1	Yes
11	Cyclohexanepropanoic acid, 2-oxo-, methyl ester	184.23	3	0	49.55	1.6989	No	No	0	Yes
12	1r,2c,3t,4t-Tetramethyl-cyclohexane	140.27	0	0	48.07	3.3246	No	No	1	Yes
13	Tridecanoic acid, 12-methyl-, methyl ester	242.40	2	0	75.50	4.7164	No	No	0	Yes
14	Neophytadiene	278.52	0	0	97.31	7.1677	No	No	1	Yes
15	Phytyl tetradecanoate	506.89	2	0	166.36	11.6161	No	No	2	**No**
16	Phytyl decanoate	450.78	2	0	147.14	10.0557	No	No	1	Yes
17	Tetradecanoic acid, 10,13-dimethyl-, methyl ester	270.45	2	0	85.12	5.3525	No	No	1	Yes
18	Ethyl 13-methyl-tetradecanoate	270.45	2	0	85.12	5.4966	No	No	1	Yes
19	n-Hexadecanoic acid	256.42	2	1	80.80	5.5523	No	No	1	Yes
20	Tetracontane-1,40-diol	595.08	2	2	196.72	13.7948	No	No	2	**No**
21	Methyl 8,11,14-heptadecatrienoate	278.43	2	0	88.50	5.3588	No	No	1	Yes
22	Bis(2-ethylhexyl) phthalate	390.56	4	0	116.30	6.433	No	No	1	Yes
23	Ethyl 15-methyl-hexadecanoate	298.50	2	0	94.73	6.2768	**Yes**	No	1	Yes
24	Tricosanal	338.61	1	0	112.88	8.3973	No	No	1	Yes
25	Phthalic acid, di(trans-dec-3-enyl) ester	442.63	4	0	134.58	7.8336	No	No	1	Yes

Abbreviations: AT, AMES toxicity; DL, drug likeness; HT, hepatotoxicity; logarithmic P, predicted octanol/water partition coefficient; MR, molecular refractivity; MW, molecular weight; NHA, number of hydrogen bond acceptor; NHD, number of hydrogen bond donor; NLV, number of Lipinski violation.

**Table 7:** Docking score (kilocalorie per mole) of different ligands with the 5NN8 and 4U14 protein.

Serial no.	Name of the ligands	**PubChem CID**	**5NN8**	**4U14**
1	(3E,7E)-4,8,12-Trimethyltrideca-1,3,7,11-tetraene	6443227	**−6.1**	**−7.0**
2	(R)-(-)-4-Methylhexanoic acid	12600623	−4.7	−5.0
3	1H-Pyrrole-2,5-dione, 3-ethyl-4-methyl-	29995	−5.3	−5.7
4	1-Propanone, 2-bromo-1-phenyl-	6931748	−5.8	−6.2
5	1r,2c,3t,4t-Tetramethyl-cyclohexane	94277	−5.8	−6.7
6	2H-Pyran, 2-(7-dodecynyloxy) tetrahydro-	86051	−5.1	−6.5
7	Bis-(2-ethylhexyl) phthalate	8343	**−6.3**	**−8.3**
8	Cyclohexanepropanoic acid, 2-oxo-, methyl ester	112036	−5.2	−6.3
9	Ethyl 13-methyl-tetradecanoate	71380066	−5.1	−5.8
10	Heptacosanoic acid, 25-methyl-, methyl ester	554101	−5.1	**−7.0**
11	Methyl 8,11,14-heptadecatrienoate	91697551	−5.8	−6.5
12	Neophytadiene	10446	−5.8	−6.2
13	n-Hexadecanoic acid	985	−4.7	−6.1
14	Phthalic acid, di(trans-dec-3-enyl) ester	91694627	**−6.3**	**−7.1**
15	Phytyl decanoate	140471960	−5.0	−5.3
16	Tetradecanoic acid, 10,13-dimethyl-, methyl ester	554145	−4.4	−5.6
17	Tricosanal	155761	−4.4	−5.5
18	Tridecanoic acid, 12-methyl-, methyl ester	21204	−4.8	−5.3
19	Acarbose	9811704	**−7.2**	
20	Loperamide	3955		**−8.3**

**Table 8:** Interaction of amino acids of selected compounds with the 4U14 protein.

**Ligands**	**Interacting amino acids**
(3E,7E)-4,8,12-Trimethyltrideca-1,3,7,11-tetraene	Ile116, Trp503, Tyr148, Ala238, Trp199, Tyr533, Tyr529, Cys532
Bis(2-ethylhexyl) phthalate	Ile116, Trp503, Tyr148, Ala235, Trp199, Tyr533, Tyr529, Cys532, Tyr506, Leu225, Leu144, Ile222, Tyr148
Heptacosanoic acid, 25-methyl-, methyl ester	Leu225, Ile222, Trp143, Leu144, Tyr506, Tyr 529, Ala238, Trp199, Tyr148, Trp503
Phthalic acid, di(trans-dec-3-enyl) ester	Tyr127, trp525, Phe221, Lys522, Trp143, Tyr529, Cys220, Leu144, Ile222, Leu225
Loperamide	Phe196, Cys111, Trp192, Ile188, Tyr104, Val149, Ala200, Ile146

**Table 9:** Interaction of amino acids of selected compounds with the 5NN8 protein.

**Name of the ligands**	**Interacting amino acid**
(3E,7E)-4,8,12-Trimethyltrideca-1,3,7,11-tetraene	Val755, Leu756, Val740, Trp804, Lys760, Val763
Bis(2-ethylhexyl) phthalate	His717, Phe362, His584, Pro595, Arg594, Val867, Leu868, Val718, Tyr360
Phthalic acid, di(trans-dec-3-enyl) ester	Glu866, Tyr360, His717, Leu868, Val867, Arg594, Arg608, Tyr609
Acarbose	Ser864, Arg608, Tyr360, Asp356, Glu866

## Data Availability

All experimental data along with raw lab data were preserved by the authors.
